# Supplementary material for: The temporal representation of experience in subjective mood
Source: eLife. 2021 Jun 15;10:e62051. doi: 10.7554/eLife.62051 (PMC8241441; doi:10.7554/eLife.62051)
Supplement: Supplementary file 1. — Note that only streaming prediction errors were used for model selection, and we show the training errors for illustrative purposes. [file elife-62051-supp1.docx]

|  |  | Training error | | Wilcoxon test | Streaming prediction error | | Wilcoxon test |
| --- | --- | --- | --- | --- | --- | --- | --- |
|  | Model | Median | iqr | p value | Median | iqr | p value |
| Recency model simulation | Primacy | 0.0067 | 0.0028 | 1.70E-06 | 0.0091 | 0.0017 | 0.0034 |
|  | Recency | 0.0062 | 0.0024 |  | 0.0083 | 0.0014 |  |
| Primacy model simulation | Primacy | 0.0059 | 0.0019 | 0.0014 | 0.0079 | 0.0022 | 6.10E-05 |
|  | Recency | 0.0067 | 0.0022 |  | 0.0096 | 0.0039 |  |
|  |  |  |  |  |  |  |  |

**Supplementary File 1:** Model parameters recovery analysis: results of fitting the Primacy and Recency models on simulated datasets as well as a statistical comparison between the two models in both the training errors and the streaming prediction errors. Note that only streaming prediction errors were used for model selection and we show the training errors for illustrative purposes.
