# Supplementary material for: The temporal representation of experience in subjective mood
Source: eLife. 2021 Jun 15;10:e62051. doi: 10.7554/eLife.62051 (PMC8241441; doi:10.7554/eLife.62051)
Supplement: Supplementary file 2. [file elife-62051-supp2.docx]

| Model name | C | E | R |
| --- | --- | --- | --- |
| Dynamic win probability | *C(t)* | $p\left( t \right)H\left( t \right)+\left( 1-p\left( t \right) \right)L\left( t \right)$ | A(t) - E(t) |
| No Certain term | None | $\frac{H\left( t \right)+L\left( t \right)}{2}$ | A(t) - E(t) |
| Outcome as expectation | *C(t)* | A(t-1) | A(t) - E(t) |
| Dynamic win probability and no Certain term | None | $p\left( t \right)H\left( t \right)+\left( 1-p\left( t \right) \right)L\left( t \right)$ | A(t) - E(t) |

**Supplementary File 2**: The formulation of alternative variants of the Recency model.
