# Supplementary material for: The temporal representation of experience in subjective mood
Source: eLife. 2021 Jun 15;10:e62051. doi: 10.7554/eLife.62051 (PMC8241441; doi:10.7554/eLife.62051)
Supplement: Transparent reporting form [file elife-62051-transrepform.pdf]

eLIFE
1st Floor
24 Hills Road
Cambridge CB2 1JP, UK
P 01223 855340
W elifesciences.org
T @elife

eLife's transparent reporting form

We encourage authors to provide detailed information *within their submission* to facilitate the interpretation and replication of experiments. Authors can upload supporting documentation to indicate the use of appropriate reporting guidelines for health-related research (see EQUATOR Network), life science research (see the BioSharing Information Resource), or the ARRIVE guidelines for reporting work involving animal research. Where applicable, authors should refer to any relevant reporting standards documents in this form.

If you have any questions, please consult our Journal Policies and/or contact us: editorial@elifesciences.org.

Sample-size estimation

- You should state whether an appropriate sample size was computed when the study was being designed
- You should state the statistical method of sample size computation and any required assumptions
- If no explicit power analysis was used, you should describe how you decided what sample (replicate) size (number) to use

Please outline where this information can be found within the submission (e.g., sections or figure legends), or explain why this information doesn't apply to your submission:

The sizes of the different samples are detailed in the Methods sections: "participants" and "Testing the Primacy model across participant characteristics and neural signals", on pages 27 and 28 respectively, as well as throughout the Results. These sizes were chosen to be necessarily larger than the minimal number of participants required to reach significant neural results when analyzing fMRI data (according to the studies titled "Estimating sample size in functional MRI (fMRI) neuroimaging studies: Statistical power analyses" and "Sample size evolution in neuroimaging research: An evaluation of highly-cited studies (1990–2012) and of latest practices (2017–2018) in high-impact journals" the lower boundary should be of 12-24 subjects). Moreover, we made sure to have a sufficient sample size to enable a leave-out sample validation when developing and fitting the different mood models (in this validation a sub-sample of 40% randomly selected participants were modelled first then followed by confirmatory analysis on the entire sample). In addition, we conducted a power analysis for the effect of mood modification in the Structured-Adaptive task, which resulted in a requirement for a sample size between 9-16 subjects to repeat a similar effect on mood (depending on the effect of interest), which was also much smaller than the sample size we have used (60-89 participants).

Replicates

- You should report how often each experiment was performed
- You should include a definition of biological versus technical replication
- The data obtained should be provided and sufficient information should be provided to indicate the number of independent biological and/or technical replicates
- If you encountered any outliers, you should describe how these were handled
- Criteria for exclusion/inclusion of data should be clearly stated

eLife Sciences Publications, Ltd is a limited liability non-profit non-stock corporation incorporated in the State of Delaware, USA, with company number 5030732, and is registered in the UK with company number FC030576 and branch number BR015634 at the address 1st Floor, 24 Hills Road, Cambridge CB2 1JP | August 2014

- High-throughput sequence data should be uploaded before submission, with a private link for reviewers provided (these are available from both GEO and ArrayExpress)

Please outline where this information can be found within the submission (e.g., sections or figure legends), or explain why this information doesn't apply to your submission:

This work included multiple replications of the main results, across three different reward environment tasks (see Methods pages 25-26), across age groups of either adolescents or adults, across healthy and depressed (see Methods page 28), as also summarized in Table S1. See page 27 (the online Mturk collection) as well as page 28 (the lab-based adolescent fMRI collection) for a description of participants exclusion.

Statistical reporting

- Statistical analysis methods should be described and justified
- Raw data should be presented in figures whenever informative to do so (typically when N per group is less than 10)
- For each experiment, you should identify the statistical tests used, exact values of N, definitions of center, methods of multiple test correction, and dispersion and precision measures (e.g., mean, median, SD, SEM, confidence intervals; and, for the major substantive results, a measure of effect size (e.g., Pearson's r, Cohen's d)
- Report exact p-values wherever possible alongside the summary statistics and 95% confidence intervals. These should be reported for all key questions and not only when the p-value is less than 0.05.

Please outline where this information can be found within the submission (e.g., sections or figure legends), or explain why this information doesn't apply to your submission:

See Methods, "Model comparison" section, page 25, for a detailed description and justification of the statistical test used to compare model performance. Additional statistical tests that were used are presented with detailed estimates in each section of the Results, as well as in the Methods sections "Statistical testing of the influence of reward environments on mood" on page 28 and "Statistical significance" on page 31.

(For large datasets, or papers with a very large number of statistical tests, you may upload a single table file with tests, Ns, etc., with reference to sections in the manuscript.)

Group allocation

- Indicate how samples were allocated into experimental groups (in the case of clinical studies, please specify allocation to treatment method); if randomization was used, please also state if restricted randomization was applied
- Indicate if masking was used during group allocation, data collection and/or data analysis

Please outline where this information can be found within the submission (e.g., sections or figure legends), or explain why this information doesn't apply to your submission:

Participants were randomly assigned to the different experimental groups, as detailed on page 27 (for the online Mturk collection) and on page 28 (for the lab-based adolescent fMRI collection).

eLife Sciences Publications, Ltd is a limited liability non-profit non-stock corporation incorporated in the State of Delaware, USA, with company number 5030732, and is registered in the UK with company number FC030576 and branch number BR015634 at the address 1st Floor, 24 Hills Road, Cambridge CB2 1JP | August 2014

eLIFE
1st Floor
24 Hills Road
Cambridge CB2 1JP, UK
P 01223 855340
W elifesciences.org
T @elife

Additional data files ("source data")

- We encourage you to upload relevant additional data files, such as numerical data that are represented as a graph in a figure, or as a summary table
- Where provided, these should be in the most useful format, and they can be uploaded as "Source data" files linked to a main figure or table
- Include model definition files including the full list of parameters used
- Include code used for data analysis (e.g., R, MatLab)
- Avoid stating that data files are "available upon request"

Please indicate the figures or tables for which source data files have been provided:

To enable the reproducibility of this study we made scripts and datasets available online at: https://osf.io/vw7sz/?view_only=e8cb4ef6782e4735815867203971994a. This online repository includes all code and source-data required to replicate the main results. Specifically, mood modeling scripts, the source-data of Figure 2 (the three tasks values and mood ratings of all participants); neural analyses scripts (the code for generating the full processing stream for each participant as well as the group level model-based correlation analysis); whole-brain neural imaging files which are presented in Figure 4.

eLife Sciences Publications, Ltd is a limited liability non-profit non-stock corporation incorporated in the State of Delaware, USA, with company number 5030732, and is registered in the UK with company number FC030576 and branch number BR015634 at the address 1st Floor, 24 Hills Road, Cambridge CB2 1JP | August 2014
